# Dehydroepiandrosterone: a potential therapeutic agent in the treatment and rehabilitation of the traumatically injured patient

**DOI:** 10.1186/s41038-019-0158-z

**Published:** 2019-08-02

**Authors:** Conor Bentley, Jon Hazeldine, Carolyn Greig, Janet Lord, Mark Foster

**Affiliations:** 1 NIHR Surgical Reconstruction and Microbiology Research Centre, University Hospital Birmingham, Birmingham, B15 2WB UK; 2 School of Sport, Exercise and Rehabilitation Sciences, University of Birmingham, Birmingham, UK; 3 MRC-Arthritis Research UK Centre for Musculoskeletal Ageing Research, Institute of Inflammation and Ageing, Birmingham University Medical School, Birmingham, B15 2TT UK; 4 NIHR Biomedical Research Centre, University Hospital Birmingham, Birmingham, UK; 5 Royal Centre for Defence Medicine, Birmingham Research Park, Birmingham, B15 2SQ UK

**Keywords:** Dehydroepiandrosterone, Dehydroepiandrosterone sulphate, Traumatic injury, Intensive care, Immune, Rehabilitation

## Abstract

Severe injuries are the major cause of death in those aged under 40, mainly due to road traffic collisions. Endocrine, metabolic and immune pathways respond to limit the tissue damage sustained and initiate wound healing, repair and regeneration mechanisms. However, depending on age and sex, the response to injury and patient prognosis differ significantly. Glucocorticoids are catabolic and immunosuppressive and are produced as part of the stress response to injury leading to an intra-adrenal shift in steroid biosynthesis at the expense of the anabolic and immune enhancing steroid hormone dehydroepiandrosterone (DHEA) and its sulphated metabolite dehydroepiandrosterone sulphate (DHEAS). The balance of these steroids after injury appears to influence outcomes in injured humans, with high cortisol: DHEAS ratio associated with increased morbidity and mortality. Animal models of trauma, sepsis, wound healing, neuroprotection and burns have all shown a reduction in pro-inflammatory cytokines, improved survival and increased resistance to pathological challenges with DHEA supplementation. Human supplementation studies, which have focused on post-menopausal females, older adults, or adrenal insufficiency have shown that restoring the cortisol: DHEAS ratio improves wound healing, mood, bone remodelling and psychological well-being. Currently, there are no DHEA or DHEAS supplementation studies in trauma patients, but we review here the evidence for this potential therapeutic agent in the treatment and rehabilitation of the severely injured patient.

## Background

Before the modern era of resuscitative medicine and surgery, and the reorganisation of emergency hospital care into major trauma centres, the likelihood of survival following a traumatic injury was down to the individual’s physiological response to injury. Survival is achieved via a complex set of metabolic, endocrine and immunological pathways [1–3] that mobilise fuel sources and minimise blood loss, so that our vital organs may continue to be perfused and function. There is growing interest in this physiological response to major trauma and how to manipulate recovery to improve patient outcomes further. That the response may be malleable and include modulation by sex steroid hormones is driven by clinical observations of lower mortality rates in females [4] and lower incidence of pneumonia post-injury compared to males [5]. Additionally, those trauma victims aged over 75 years have an increased morbidity and mortality rate [6]. The differences in sex steroid hormones and their precursors between genders and across the lifespan may thus influence outcomes in the trauma patient population [7].

## Review

### The endocrine, immune and metabolic response to trauma

**Fig. 1:**
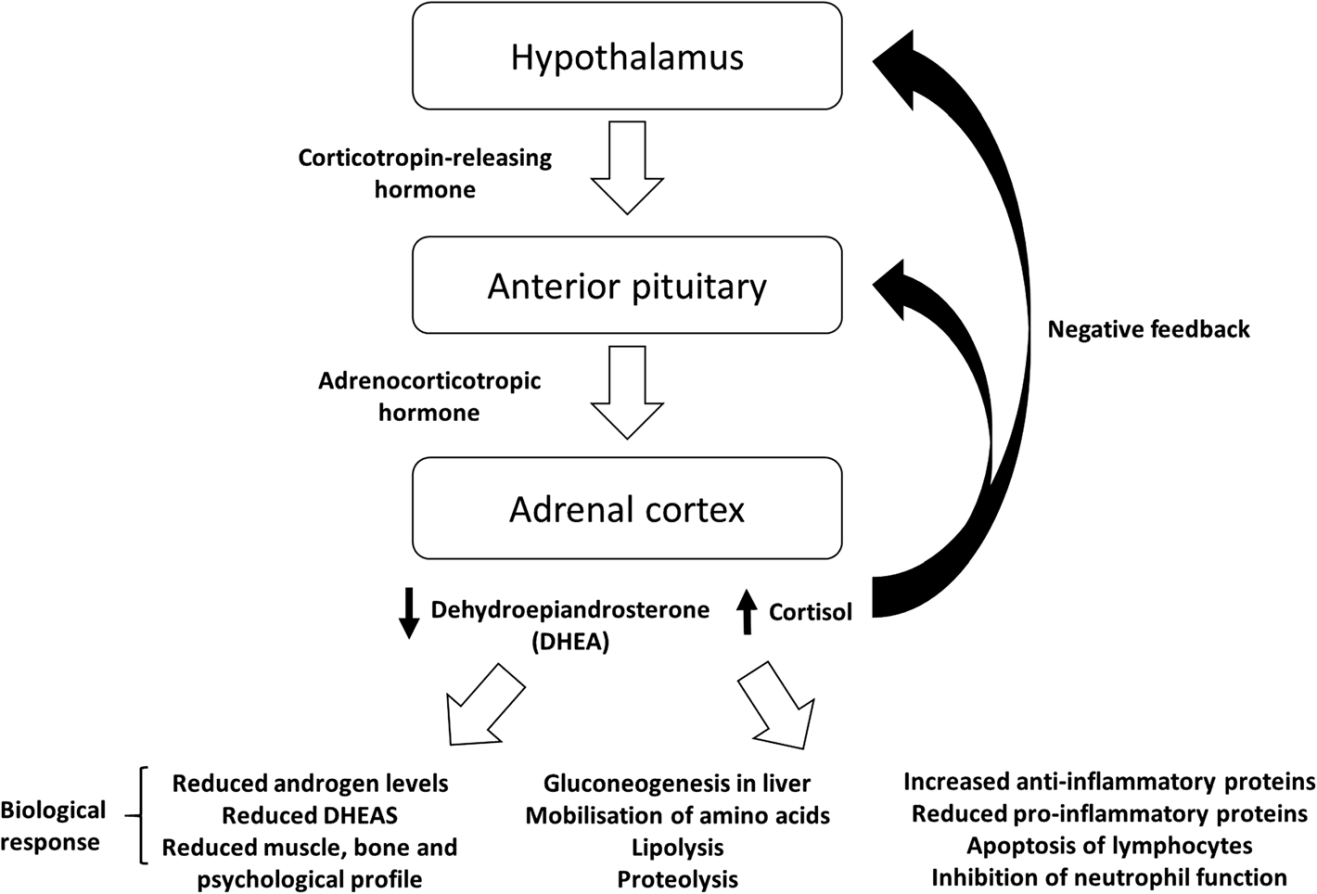
The hypothalamic–pituitary–adrenal (HPA) axis and the biological responses that occur as a result of traumatic injury. The resulting increase in circulating levels of cortisol and a reduction in dehydroepiandrosterone (DHEA) has been shown to affect several biological responses, such as the inhibition of neutrophil function

As previously reviewed [1, 3], the metabolic and endocrine response to trauma is mediated through the hypothalamus. Via baro-, volu- and pain receptors, the hypothalamus receives multiple signals as a result of the injury, which initiates the acute phase response and activation of the pituitary, adrenal and sympathetic nervous system pathways, which induces the characteristic ‘fight or flight’ response. Central to this response is cortisol (Fig. 1).

Cortisol is vital in the immediate response to trauma as it increases blood glucose via protein catabolism, promoting hepatic gluconeogenesis and allowing for an increase in gluconeogenic precursors from triglyceride breakdown. Cortisol also acts upon cells of the immune system, including macrophages [8] and neutrophils, preventing their excessive accumulation in damaged tissues and areas of inflammation but also potentially limiting the anti-microbial effects by inhibiting their function [9]. In addition to inhibition of cytokine production, cortisol decreases the production of pro-inflammatory leukotrienes and prostaglandins [10].

The hypothalamic–pituitary axis should, in theory, receive negative feedback from cortisol to prevent further release of corticotropin-releasing hormone (CRH) and adrenocorticotropic hormone (ACTH) [1, 3]. A second adrenal steroid hormone affected by trauma is dehydroepiandrosterone (DHEA), an androgen precursor, which is mostly present in the circulation in its sulphated form, dehydroepiandrosterone sulphate (DHEAS). After injury, serum DHEAS levels fall, and the synthesis of cortisol overtakes that of DHEAS [11], which is plausibly due to inhibition of DHEA sulphation, which is down regulated in acute inflammation and sepsis [12].

Initiated via blood loss and tissue damage, damage-associated molecular patterns (DAMPs), such as high-mobility group box (HMGB)-1 [13], are secreted from activated neutrophils [14] and necrotic cells [15]. Necrotic cells evoke a strong reaction due to the release of mitochondrial DNA and cellular constituents such as formylated peptides [16, 17]. DAMPs can directly activate neutrophils and monocytes via specific DAMP receptors [14], with activation of both C3a and C5a complement [18] synergistically causing the production and subsequent release of interleukins thereby generating the systemic inflammatory response syndrome (SIRS) [2].

The SIRS response is accompanied by a compensatory anti-inflammatory response syndrome (CARS) [19], to restore homeostasis. If the resolution of inflammation is not achieved via anti-inflammatory cytokines, CARS may progress to the persistent inflammation, immunosuppression and catabolism syndrome (PICS) [20, 21]. Compounding the problems that have arisen from the initial injury, the PICS clinical picture may involve: reduced oxygen and nutrient delivery via macro and microcirculatory impairment [20]; acquired weakness in the intensive care unit (ICU) [22] and a subsequent dependence on mechanical ventilation; muscle atrophy, which is related to increased multi-organ failure [23]; and increased sepsis [24].

Therefore, a clinically driven research solution to promote a shorter period in catabolism, more rapid return to anabolism and the recovery of immune function is required if we are to improve patient outcomes following significant injury.

### DHEA/DHEAS and the response to trauma

Differences between the sexes in response to injury have been observed. Being male and a victim of trauma is associated with increased mortality, length of hospital stay and secondary complications such as infections and multiple organ failure. This suggests that the female sex steroid hormones may have a protective effect in trauma [7, 25]. The apparent female advantage [26] and differences in survival with age [27] have led to the consideration of the role of the sex steroid precursor hormone DHEA, whose levels decline with age and differ between the sexes.

DHEA is predominantly synthesised in the zona reticularis of the adrenal cortex [28] in response to stimulation by ACTH. Both DHEA and DHEAS are secreted from the adrenal cortex, with peak concentrations of 10 μM (DHEAS) and 10 nM (DHEA). In humans, serum levels of both DHEA and DHEAS change significantly across the lifespan [29]. Large amounts are produced during fetal development and after an initial rapid decline immediately after birth [30] synthesis resumes when adrenarche occurs between 6 and 8 years [31]. DHEA and DHEAS levels continue to rise throughout puberty and peak during the second decade of life with absolute levels of circulating DHEAS lower in females than males throughout life [32]. By the third decade of life, DHEA and DHEAS levels decline [33], being as little as 10–20% of peak by the eighth decade [34], a phenomenon termed the adrenopause.

**Fig. 2:**
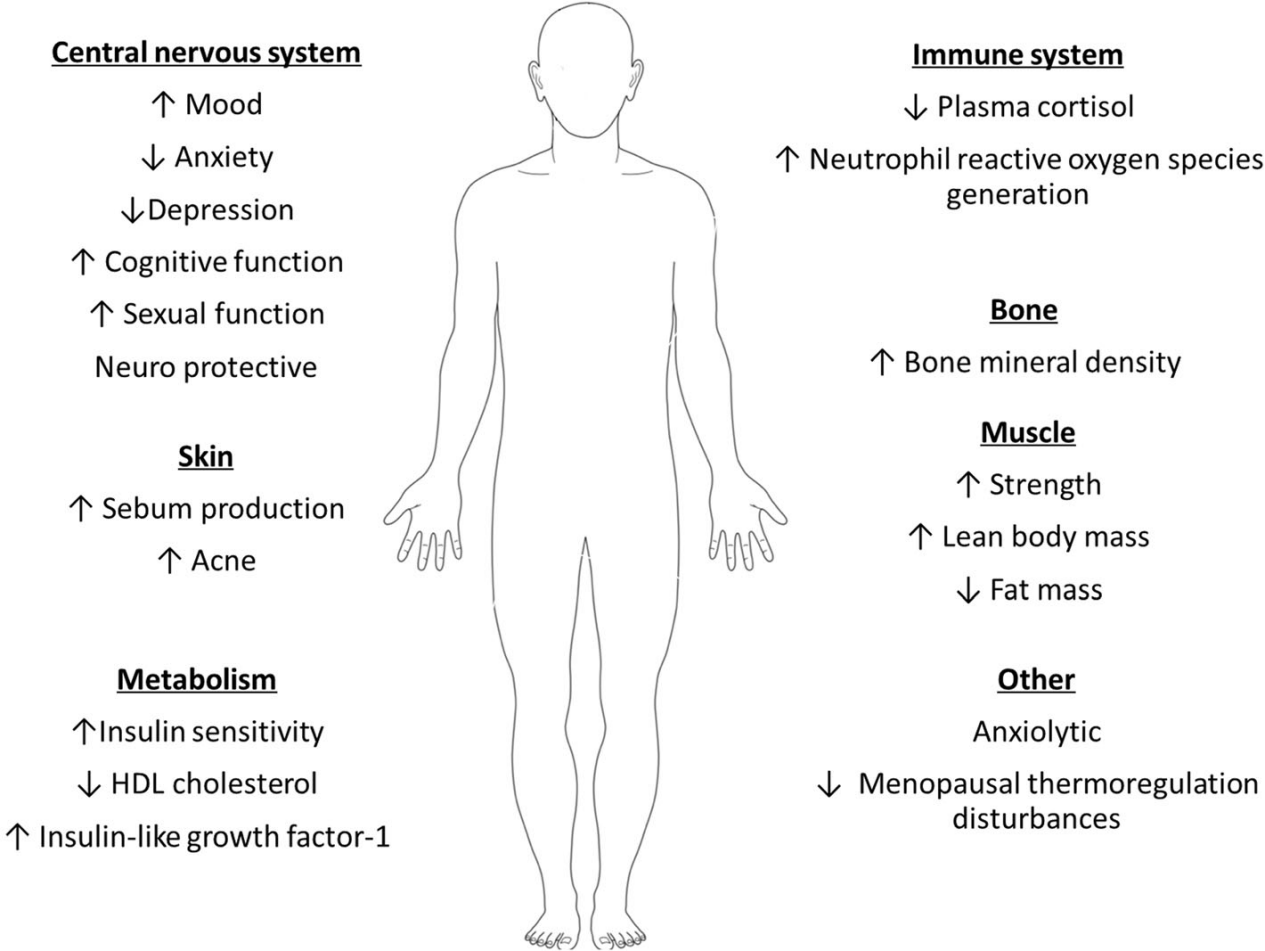
The physiological effects of dehydroepiandrosterone/dehydroepiandrosterone sulphate (DHEA/DHEAS) in humans. Restoration to normal DHEA and DHEAS levels are associated with a range of positive biological functions such as, increased (↑) bone mineral density and decreased (↓) fat mass, across a range of body systems that include the bone and central nervous system (CNS), high density lipoprotein (HDL)

DHEA is sulfonated by the sulfotransferase family 2A member 1 (SULT2A1) to DHEAS [35] in the adrenal cortex and during first-pass metabolism in the liver and, as such, would have implications upon oral DHEA supplementation. The levels of circulating DHEAS are several folds higher than DHEA [36], acting as a reserve to be readily converted by steroid sulfatase (STS) to DHEA in the endoplasmic reticulum [37, 38]. Only 5% of DHEA in males with normal testicular function is converted to testosterone [39]. However, in premenopausal females, 40–75% of the circulating testosterone comes from DHEAS. This is in stark contrast to the 90% of oestrogens that are derived from DHEAS in the postmenopausal female [39]. Some of the known biological functions of DHEA are shown in Fig. 2.

DHEA has been shown to modulate the action of glucocorticoids, such as cortisol [40]. We have reviewed the numerous immune effects of DHEA and DHEAS previously [41] and have shown that DHEAS but not DHEA enhances neutrophil superoxide generation via protein kinase C (PKC) mediated pathway, thereby augmenting an essential immune response to infection [42]. We and others have also suggested an influence of DHEA/DHEAS on anti-viral innate immune function. In Addison’s disease, in which autoantibodies target the adrenal cortex, patients are supplemented with sex steroids and glucocorticoids but not DHEA/DHEAS. These patients have reduced natural killer (NK) cell cytotoxic function [43] and increased susceptibility to respiratory infections [44]. These patients also show a reduced innate anti-viral response in peripheral blood mononuclear cells, specifically reduced chemokine (C-X-C motif) ligand (CXCL)9 and CXCL10 production in response to stimulation with interferon [45]. However, supplementation with DHEA did not improve NK cell function in these patients [43].

Cortisol levels are preserved with age and, as mentioned previously, are elevated after severe injury, as well as sepsis [24]. In addition to being potent suppressors of the immune system, glucocorticoids promote an intra-adrenal shift in steroid biosynthesis, being produced at the expense of DHEAS [46]. This increases the cortisol to DHEAS ratio which is associated with a range of poor outcomes in trauma victims [47–51], with further exacerbation of this ratio in those with adrenopause, associated with suppressed neutrophil function and increased risk of infections [9].

Our group has shown in a 6-month observational study of major trauma patients that DHEAS levels were reduced within days of injury to almost undetectable levels. DHEAS remained low throughout the follow-up period, despite DHEA returning to normal by 3 months. Resolution of the DHEA: cortisol ratio to that of healthy controls provides an insight into potential clinical benefits, namely, a decrease in nitrogen excretion and increase in biceps brachii muscle thickness, suggesting a reversal of catabolism with the normalisation of DHEA levels [52, 53]. The DHEAS: cortisol ratio is proposed to represent a balance between the catabolic effects of cortisol and the regenerative effects of DHEAS [54, 55] and its modulation may benefit the trauma patient. Unlike DHEAS, DHEA is readily available in various formulations. As a result, the vast majority of clinical studies have employed DHEA supplementation as a route to influencing this ratio and our review, therefore, focusses on this intervention.

### DHEA and DHEAS deficiency after trauma and critical illness

Twenty-two studies investigating the level of DHEA and DHEAS after critical illness have been identified by our group [9, 11, 12, 56–74]. Only two studies have measured DHEA [72, 74] via the gold standard method of liquid-chromatography mass-spectroscopy (LC-MS) [75] in traumatically injured patient. Although Brorsson et al. [72] demonstrated a significant decrease in both DHEA and DHEAS levels within the study duration of 96 h, these short-term follow-up studies with often mixed clinical populations, make direct comparisons to the young and non-septic trauma populations unclear. Foster et al. study in 102 severely injured patients, 41 of whom were young male soldiers, identified that in addition to low DHEA and DHEAS levels for up to 6 months post-injury, the downstream suppression of androgens highlights an opportunity to intervene in adrenal androgen synthesis in the medium to longer term recovery after both battlefield and civilian trauma [53]. Despite its potential, interventional studies have yet to be designed to address both DHEAS and DHEA's ratio with cortisol in traumatically injured patients.

### The benefits of DHEA and DHEAS in recovery after trauma

To our knowledge, no human studies have utilised DHEA supplementation in trauma patients at any stage of their recovery, despite being proposed [34]. Similarly, DHEAS has yet to be used as an intervention in any human trauma studies. It may be that DHEAS supplementation would be more beneficial than DHEA in potentiating the immune function, such as enhancing reactive oxygen species (ROS) production by neutrophils via activation of nicotinamide adenine dinucleotide phosphate (NADPH) oxidase [76]. However, what we do know is that supplementation of DHEA in healthy subjects, via oral administration, will result in first-pass metabolism and thus a conversion of DHEA to DHEAS, resulting in either immune or downstream androgen or oestrogenic benefits.

Animal models have demonstrated numerous benefits from DHEA supplementation such as improved hyperglycaemia [77], decreased mortality after trauma-induced haemorrhage [78], neurogenesis [79] and wound reperfusion [80], all of which pose a considerable burden to the recovery from injury. It is important to note that rodent adrenal DHEA production is modest [81]. Rodents possess the necessary mechanisms to convert exogenous DHEA to sex steroids [82], but caution is required in extrapolating rodent data to humans.

#### Inflammation and immune effects

A retrospective analysis of the trauma register from 2002 to 2005, reported bilateral femoral shaft fractures, which are often accompanied with abdominal injuries and blood loss, as being an independent risk factor for pulmonary failure [83]. A mouse model was used by Lichte and colleagues to confer if subcutaneous DHEA administration (25 mg/kg/day) would control the systemic inflammation seen in the treatment of these injuries. Replicating the musculoskeletal damage that is observed in a bilateral femoral fracture, DHEA supplemented mice benefited from a reduction in serum tumour necrosis factor (TNF)-α, interleukin (IL)-1β, IL-6, IL-10, monocyte chemoattractant protein (MCP)-1. However, DHEA did not improve markers of pulmonary inflammation [84].

Animal and human studies indicate that steroid hormones influence cellular immunity. The reversal of trauma-induced suppression of splenocyte proliferation, macrophage TNF-α, IL-1 and IL-6 production [78, 85] and IL-2, IL-3 and interferon (IFN)-γ secretion from splenic T cells [85, 86], improved mortality rates [78] and prevention of increased serum corticosterone [86] have all been observed following the administration of a single subcutaneous injection of DHEA in rodent models of traumatic haemorrhage. Additionally, Oberbeck et al. demonstrated that DHEA supplementation normalised splenocyte apoptosis and lymphocyte migration in haemorrhagic shock [87]. In mice models of sepsis, a frequent complication in the recovering trauma patient, the administration of DHEA has been shown to improve survival [87]. Mouse models of thermal injury have demonstrated increased resistance to pathogenic challenges when compared to controls [88] and reduced inflammation and tissue necrosis [89] when subcutaneous DHEA was administered. Conversely, other forms of steroids, including DHEAS, exhibited no protective effects [89].

There are very few studies on the effects of DHEA or DHEAS on human immune cells. DHEAS has been shown to directly stimulate the action of NADPH oxidase and reactive oxygen species production and thus improve neutrophil function [42]. This effect may be unique to neutrophils as these immune cells are the only leukocytes that express the organic anion transporting polypeptide (OATP-D) required for DHEAS uptake. The very low levels of DHEAS seen after trauma may thus be a contributor to reduced neutrophil function in trauma patients [90]. In contrast, in the hyperglycaemic environment that is often present after trauma and infection, DHEA, a glucose-6-phosphate dehydrogenase inhibitor, has been shown to reduce neutrophil superoxide production in a dose-dependent manner [91].

Recently, Corsini et al. have identified DHEA conversion to androgens and subsequent binding to androgen receptors, as a necessary step in the DHEA-induced monocyte activation and its potential use for immune modulation [92]. DHEA may also prevent monocyte adhesion in endothelial cells, appearing to act via its oestradiol and dihydrotestosterone metabolites [93]. This conversion of DHEA to downstream metabolites may occur within macrophages, which may also be important for local immunomodulation, although the conversion does depend upon the maturation of the monocyte to a macrophage in tissues [94].

#### Wound healing

Trauma results in physical injury which may be acute (a result of the initial trauma) or chronic (due to impaired wound healing). Wound healing begins immediately after the injury and involves a series of overlapping phases with the sequential recruitment of immune cells, fibroblasts, stem cells and endothelial cells to mediate tissue repair and extracellular matrix deposition [95, 96].

Advances in surgical techniques have led to the rise in the use of tissue flaps to improve the outward appearance and functionality that has been lost. Rats pre-treated with DHEA had markedly improved muscle flap microcirculation and haemodynamics and were protected against ischaemia and reperfusion injury [80]. A similar study by Ayhan and colleagues supplemented rats with intravenous DHEA and showed a reduction in activation of leukocytes, improved red blood cell velocity and capillary perfusion in the muscle flap microcirculation, with the protective effect most likely a result of delayed expression of Mac-1 integrin, L-selectin and CD44 molecules on leukocytes [97]. Topical administration of DHEA has also acted as a mediator of tissue repair to ultra violet (UV) light damaged skin [98].

How DHEA exerts its effects on wound healing are not precisely known. Excessive inflammation is a causative factor in delayed wound healing, and DHEA supplementation inhibits nuclear factor kappa-light-chain-enhancer of activated B cells (NF-κB) DNA binding activity, resulting in the dampening of gene transcription of IL-6 and TNF-α [99]. Alternatively, it may be DHEA conversion to both androgens and oestrogens that is involved, as sex hormones have also been shown to be anti-inflammatory [100, 101]. In a mouse model of age-related delayed wound healing, Mills et al. observed that topical administration of DHEA accelerated wound healing, dampened the inflammatory response via mitogen-activated protein kinase (MAPK) and phosphatidylinositol 3 (PI3) kinase pathways. The authors suggested this beneficial response was due to DHEA conversion to oestrogen [102].

Interestingly, animal models of wound healing have shown that DHEA has no observable effects in the young. This is possibly due to circulating oestrogens being sufficient in the young, or supra-physiologically high levels of oestrogen via DHEA conversion exerting no effect upon wound healing [102]. Although this suggests that topical DHEA may benefit the older trauma patient, there is currently insufficient data to suggest routine administration for wound healing, across all trauma demographics.

#### Psychological and neurological effects

Traumatic brain injury (TBI) is a major cause of mortality and morbidity, with 1.4 million patients per year attending hospital in England and Wales [103]. DHEA and DHEAS may be synthesised by the brain independently with levels higher in the central nervous system (CNS) when compared with blood [104, 105]. In rats, DHEA supplementation (40 mg/kg for 5 days subcutaneously) has been shown to promote neurogenesis in the hippocampus and survival of newly formed neurons [79]. Additionally, supplementation of a DHEA analogue has been shown to improve cognitive and motor skills via a beam walk test in TBI rats, with authors hypothesising the action via retardation of glial scar formation and neurite regrowth helping to restore reflexes and memory [106]. Hoffman and colleagues observed similar results, albeit that the DHEA administration was delayed until 7 days post-injury, with improvements in a battery of behavioural tests [107]. This benefit of DHEA directly may be via neural stem cells, increased nerve growth factor (NGF) and brain-derived neurotrophic factor, potentially conferring neurogenic, neuronal survive advantages [108, 109]. Again, no studies to date have investigated the use of DHEA supplementation in a human neurotrauma cohort, with this relatively cheap supplement, potentially providing a safe medium to long-term aid to recovery and possibly pain perception [110] in these patients.

The psychological aspect of trauma is often overlooked in recovery; with an incidence of post-traumatic stress disorder (PTSD) approximately 10% in those who have been subjected to trauma [111] and depressive symptoms in 42% of patients, issues may occur any time from 6 weeks up until 20 years post-injury [112, 113]. The DHEAS: cortisol ratio predicted stress resilience in male military personnel; lower symptoms of PTSD and depression and finally demonstrating improving symptoms over time [114]. A raised cortisol: DHEAS ratio in old hip fracture patients was associated with the development of depression after injury [49], thereby detrimentally impacting the individual during a period of immune and psychological vulnerability. A 15-year follow-up in a population of Vietnam veterans, both age-adjusted and fully adjusted analysis, showed that both cortisol and the cortisol: DHEAS ratio were positively associated with hypertension [48]. As a result, both studies suggest the need to maintain an adequate DHEAS: cortisol ratio for the prevention of long-term ill health after stressful events.

DHEA levels are also inversely correlated with depressive symptoms in adults under 64 years [115], with higher serum DHEAS levels in older adults shown to be protective against the onset of depression [116]. Unfortunately, in the healthy older population, DHEA supplementation has not proven beneficial for well-being and depressive symptoms [117]. DHEA supplementation in those with moderate depression [118] and psychiatric disorders [119–121] and adrenal insufficient women [122] has shown potential for mental health. In older men, a relatively low dose of 25 mg of DHEA per day showed improvements in joint pain, hormonal profile and clinical status [123]. A 6-month DHEA supplementation of males and females with profound androgen deficiency noted minor to modest improvement in psychological well-being [124]. However, it is when DHEA is converted to DHEAS that a greater impact upon the gamma-aminobutyric acid (GABA) [125] and the N-methyl-D-aspartate (NMDA) [126] receptors may result in a psychological benefit.

#### Body composition

Several studies have suggested a positive effect of DHEA on bone biology. Wang and colleagues treated ovariectomised mice with DHEA and observed a significantly increased bone cancellous compared with that of control, suggesting that DHEA can improve bone tissue morphometry of this postmenopausal model [127]. In a further sub-study with the calvariae of neonatal mice, the authors proposed that DHEA increases the anabolic metabolism-related organelle content in osteoblasts, improving mechanical strength [127]. A recent study investigating the effect of a *Brucella abortus* infection on mouse osteoblast function showed that DHEA treatment reversed the effect of the infection upon osteoblasts by increasing their proliferation, inhibiting apoptosis and restoring differentiation and function [128].

In postmenopausal women, DHEA given orally increased bone mineral density in both the lumbar spine and femoral neck [129], which may be attributable to the conversion of DHEA to both oestrogens and active androgens in bone [130]. DHEA appears to promote the proliferation and inhibition of osteoblasts via the MAPK signalling pathway independent of an androgen or oestrogen receptor, thus suggesting the supplementation may directly exert its effect via a specific DHEA receptor [131]. Additionally, the osteoanabolic action of DHEA may act upon the adrenally insufficient patient by increasing levels of osteocalcin and bone mineral density (BMD) [132–134]. A 6-month 50 mg DHEA supplementation in older males with low DHEAS levels improved total body BMD [135]. Improved BMD was also observed in osteoporotic patients when administered 100 mg per day of DHEAS over the same period [136]. In 225 women, aged 55–85, who were supplemented with 50 mg per day for 1 year, a positive effect upon the lumbar spine BMD was observed [137]. Jankowski and colleagues also investigated the effect of DHEA supplementation upon those with low levels of DHEAS. Their study of 70 males and 70 females demonstrated significant improvements when compared to placebo, for an increase in hip BMD in both sexes and spine BMD in females [138]. As traumatic injuries and hip fractures are comprised of characteristic long bone and soft tissue injury, the potential effect of DHEA as a means of improving a patient’s bone profile in recovery may be advantageous.

The loss of muscle mass and function with age, sarcopenia, has also been targeted by DHEA. However, the effect upon males has been variable, from a minimal effect on muscle mass [139] to reduced body fat and increasing muscle strength after 1 year of DHEA supplementation [140]. A 6.1% reduction in fat mass in healthy 50–65-year-old males, with significant improvement in measures of muscle strength of the knee (15%) and lumbar strength (13.9%), has been reported with 6 months of DHEA [141]. DHEA was also able to reduce both visceral and abdominal fat while improving insulin sensitivity [141]. These beneficial effects upon insulin sensitivity may be mediated by increased oestrogen and androgen levels, but also independently, by an increase in Insulin-like growth factor (IGF)-1 [142], an important anabolic hormone. Studies in adrenal insufficiency, which may mimic the low levels of DHEA and DHEAS seen after trauma, did not reveal any significant effect of DHEA replacement on body composition, or bone parameters [132–134, 143, 144]. The rapid and prolonged loss of muscle mass, as well as low levels of DHEA, increase the risk sepsis in trauma populations [52, 53]. Therefore, DHEA supplementation may improve both the size and strength of the rehabilitating patient as well as helping to support organ function during acute illness especially as a result of ICU-acquired weakness.

#### Benefits of DHEA over up and downstream sex hormone supplementation

DHEA is the precursor for the androgenic and oestrogenic hormones. It is unlikely that supplementation of 17α-hydroxypregnenolone or pregnenolone (upstream steroid hormones) would be of benefit as both may be converted to cortisol during the stress response. After the initial acute phase of injury, when cortisol is essential, prolonged hypercortisolaemia in the weeks after injury may initiate immunosuppression by exacerbating the trauma-induced increase in the circulating concentration of this glucocorticoid [145].

Downstream androgens, such as testosterone, have been considered. Driven by the need to overcome liver toxicity of exogenous testosterone supplementation, the analogue oxandrolone has been used in both adults [146] and children [147]. Although its use has been unproven in the first month after trauma in a mixed population [148], it is in burn injury, where it has been seen to act positively to mitigate the hypermetabolism and catabolism observed in those who have a total burns surface area of > 20%, without side effects [149]. Oxandrolone still requires well-designed optimal dose-finding studies to be undertaken in the heterogeneous trauma population so that the efficacy and safety of this drug may be put through the rigour of a larger multi-centre randomised controlled trial [150], over an effective duration, in order to overcome the hypermetabolic and catabolic state observed after injury across a range of demographics [52, 53].

In summary, DHEA has potential advantages over supplementation with downstream hormones. As the precursor for the androgenic and oestrogenic hormones, it can benefit male and female patients, and unlike oxandrolone, it may be converted to DHEAS which we have previously shown to enhance neutrophil function and thus potentially offer protection against infection [9, 42].

### Practicalities: dosing, delivery and safety of DHEA supplementation studies

#### Consideration and challenges in a trauma cohort

Studies investigating the dosing and delivery routes of administration of DHEA supplementation trauma patients are fraught with difficulties. As patients will be critically ill, there is a high likelihood that they will be subjected to polypharmacy. This may adversely affect the true profiling of DHEA. Nevertheless, there are some key factors to consider for any DHEA supplementation study.

Due to DHEA metabolism in the liver, any patients with pre-existing or chronic liver failure should not be recruited; known thromboembolic events in the last 12 months and any pre-disposition to thrombosis are also contraindicated, as DHEA’s androgenic potential may result in altered coagulation [151]. As patients clinical therapy may involve the administration of drugs and blood products to counteract any blood loss or long-term admission, daily monitoring of changes to document adverse events are necessary. Patients taking hormone replacement therapy, antipsychotic medication and with known hypersensitivity to DHEA [152], should be excluded. Similarly, those who have known or previous hormone-sensitive malignancies or invasive cancer, and prostatic hypertrophy due to DHEA supplementation increasing downstream metabolites in both males [153] and females [154] may need to be excluded. Although women with poor oocyte production have been shown to benefit positively from DHEA supplementation of 50 mg/day [155], the recruitment of pregnant trauma patients would be inadvisable due to the lack of available data.

Concurrent DHEA and testosterone therapy have not been tested, but available data suggest the avoidance of such an approach [152], due to oral DHEA increasing endogenous testosterone [156]. Therefore, intake of any drugs influencing the metabolism of steroids in the months before injury should act as an exclusion criterion for potential patients [157]. Administration of progesterone and DHEA may also yield false-positive results when it comes to using commercially available progesterone assays [158], which may be overcome by using LC-MS [75].

Maxillo-facial injuries or a non-functioning gut may prohibit sublingual or oral administration and compliance to the study protocol; therefore, an adaptable study design is needed to generate pilot data. By monitoring gastric residual volumes (GRV), a surrogate marker of gastrointestinal motility [159], and seeking expert statistical advice on overcoming potential trial pitfalls, identification of an appropriate dose and route of DHEA may be identified for use in a randomised controlled trial.

#### Current DHEA therapeutic indications, investigated doses and method of administration

Researchers have attempted to circumvent first-pass metabolism by providing DHEA supplementation via different formulations and routes. Transvaginal [160], transdermal [161], subcutaneous pellets [162] and buccal/sublingual [163, 164] routes have all been investigated and shown to increase the DHEA: DHEAS ratio. Oral and buccal/sublingual delivery provide the most broadly acceptable and practical route for self-medicating. However, for any future clinical trials, the method of delivery must be one that is not only efficacious but feasible given the injuries sustained.

A DHEA buccal dose of 50 mg can increase free testosterone, total testosterone, androstenedione and DHEAS levels, with 5 mg shown to double DHEAS within 5–10 h, prior to returning to the pre-treatment levels within 24 h [163]. However, in the most investigated cohort, peri-menopausal females, 90% of studied doses have been at < 50 mg/day given orally [165]. Previous studies have shown that supplementation with 50 mg DHEA orally once daily in the older population restores serum DHEA and DHEAS levels to that of men and women in the third decade of life [166, 167]. As expression and activity of steroid metabolising enzymes in peripheral target tissues, which may be influenced by inflammatory cytokines [38], we cannot assume that downstream conversion of DHEA in patients with acute trauma and associated inflammatory response will be identical to that in healthy volunteers. Thus, the pharmacological profile of DHEA supplementation via different routes, at different doses in recovering trauma patients requires investigation via an innovative study design.

#### Possible side effects of DHEA supplementation

Cochrane reviews of DHEA supplementation in peri- and post-menopausal females [165], systemic lupus erythematosus [168], assisted reproduction [169] and older adults [117] have attempted to assess its safety. Although DHEA has been used at different doses with no deleterious side effects, longer-term studies have reported mild cases of hirsutism and acne [170–172]. The highest daily dose reported to date, in both males and females, was 1600 mg/day for 28 days [173]. Nair and colleagues undertook the longest placebo-controlled, randomised, double-blind study in older men (75 mg/day) and women (50 mg/day) for 2 years. The authors monitored prostate volume, prostate-specific antigen, liver function, electrolyte levels and haemoglobin and did not observe any significant differences between the groups [174].

**Fig. 3:**
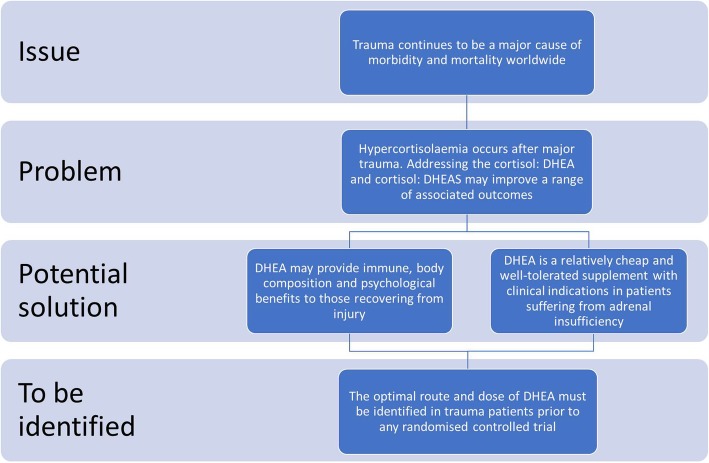
Dehydroepiandrosterone/dehydroepiandrosterone sulphate (DHEA/DHEAS): a potential therapy to the burden of traumatic injury? A schematic diagram identifying if this safe, well-tolerated, early sex-steroid could be added to the future care package of those who have been injured? Well-designed studies are needed first to identify the dose and the route of administration, prior to any large multicentre, randomised controlled trial

From the published literature, we could only find four publications that have utilised the sublingual and/or oral route for administration of DHEA. The studies used different daily doses of DHEA varying from 10 to 50 mg and were carried out for 2 weeks to 4 months [163, 164, 175, 176]. In only one of these four studies [164] was mild acne reported as a side effect of supplementation. The current data suggest that DHEA, certainly in short-term supplementation, should be regarded as safe without significant side effects. However, the outcomes, dose and administration route of DHEA supplementation require confirmation via feasibility and pilot studies in those with traumatic injury (Fig. 3).

## Conclusions

The endocrine response to traumatic injury has been well studied. However, the role of DHEA and DHEAS in severe clinical trauma is relatively unexplored, with the majority of studies focusing upon the cortisol responses. This is despite recent data suggesting that it is the cortisol to DHEAS ratio that is the superior prognostic factor for short and long-term outcomes. Results have indicated that levels of the early sex-steroid hormones, DHEA and DHEAS, are low immediately after injury and remain below normal for over 6 weeks and longer, particularly in the older patient. Animal models and previous reviews have hypothesised that exogenous supplementation of DHEA is warranted. The immunological, anabolic, neurocognitive, wound and mood enhancing profile reported in animal models, and also some human studies, provide an opportunity to support trauma patient recovery holistically. However, there is a need for studies in the trauma population to first identify the dose, route and duration of DHEA supplementation that would restore levels to those of the healthy adult.

## Abbreviations

ACTH: Adrenocorticotropic hormone
BMD: Bone mineral density
CARS: Compensatory anti-inflammatory response syndrome
CNS: Central nervous system
CRH: Corticotropin-releasing hormone
CXCL: Chemokine (C-X-C motif) ligand
DAMPs: Damage-associated molecular patterns
DHEA: Dehydroepiandrosterone
DHEAS: Dehydroepiandrosterone sulphate
HDL: High density lipoprotein
GABA: Gamma-aminobutyric acid
HMGB: High-mobility group box
ICU: Intensive care unit
IFN: Interferon
IGF: Insulin-like growth factor
IL: Interleukin
LC-MS: Liquid-chromatography mass-spectroscopy
MAPK: Mitogen-activated protein kinase
MCP: Monocyte chemoattractant protein
NADPH: Nicotinamide adenine dinucleotide phosphate
NF-κB: Nuclear factor kappa-light-chain-enhancer of activated B cells
NGF: Nerve growth factor
NK: Natural killer
NMDA: N-methyl-D-aspartate
OATP-D: Organic anion transporting polypeptide
PI3: Phosphatidylinositol 3
PICS: Persistent inflammation, immunosuppression and catabolism syndrome
PKC: Protein kinase C
PTSD: Post-traumatic stress disorder
ROS: Reactive oxygen species
STS: Steroid sulfatase
SIRS: Systemic inflammatory response syndrome
SULT2A1: Sulfotransferase family 2A member 1
TBI: Traumatic brain injury
TNF: Tumour necrosis factor
UV: Ultra-violet

## References

[1] Hastings J, Krepska A, Roodenburg O (2014). The metabolic and endocrine response to trauma. Anaesth Intensive Care.

[2] Lord JM, Midwinter MJ, Chen Y-F, Belli A, Brohi K, Kovacs EJ (2014). The systemic immune response to trauma: an overview of pathophysiology and treatment. Lancet.

[3] Krepska A, Hastings J, Roodenburg O (2017). The metabolic and endocrine response to trauma. Anaesth Intensive Care.

[4] Haider AH, Crompton JG, Oyetunji T, Stevens KA, Efron DT, Kieninger AN (2009). Females have fewer complications and lower mortality following trauma than similarly injured males: a risk adjusted analysis of adults in the National Trauma Data Bank. Surgery.

[5] Gannon CJ, Pasquale M, Tracy JK, McCarter RJ, Napolitano LM (2004). Male gender is associated with increased risk for postinjury pneumonia. Shock.

[6] Braun BJ, Holstein J, Fritz T, Veith NT, Herath S, Mörsdorf P (2016). Polytrauma in the elderly: a review. EFORT Open Rev.

[7] Al-Tarrah K, Moiemen N, Lord JM (2017). The influence of sex steroid hormones on the response to trauma and burn injury. Burns Trauma BioMed Central.

[8] Roger T, Chanson A-L, Knaup-Reymond M, Calandra T (2005). Macrophage migration inhibitory factor promotes innate immune responses by suppressing glucocorticoid-induced expression of mitogen-activated protein kinase phosphatase-1. Eur J Immunol.

[9] Ronchetti Simona, Ricci Erika, Migliorati Graziella, Gentili Marco, Riccardi Carlo (2018). How Glucocorticoids Affect the Neutrophil Life. International Journal of Molecular Sciences.

[10] Sorrells SF, Sapolsky RM (2007). An inflammatory review of glucocorticoid actions in the CNS. Brain Behav Immun.

[11] Ilias I, Stamoulis K, Armaganidis A, Lyberopoulos P, Tzanela M, Orfanos S (2007). Contribution of endocrine parameters in predicting outcome of multiple trauma patients in an intensive care unit. Hormones.

[12] Arlt W, Hammer F, Sanning P, Butcher SK, Lord JM, Allolio B (2006). Dissociation of serum dehydroepiandrosterone and dehydroepiandrosterone sulfate in septic shock. J Clin Endocrinol Metab.

[13] Pugin J (2012). How tissue injury alarms the immune system and causes a systemic inflammatory response syndrome. Ann Intensive Care.

[14] Manson J, Thiemermann C, Brohi K (2012). Trauma alarmins as activators of damage-induced inflammation. Br J Surg.

[15] Scaffidi P, Misteli T, Bianchi ME (2002). Release of chromatin protein HMGB1 by necrotic cells triggers inflammation. Nature.

[16] Zhang Q, Raoof M, Chen Y, Sumi Y, Sursal T, Junger W (2010). Circulating mitochondrial DAMPs cause inflammatory responses to injury. Nature.

[17] Kaczmarek E, Hauser CJ, Kwon WY, Riça I, Chen L, Sandler N (2018). A subset of five human mitochondrial formyl peptides mimics bacterial peptides and functionally deactivates human neutrophils. J Trauma Acute Care.

[18] Burk A-M, Martin M, Flierl MA, Rittirsch D, Helm M, Lampl L (2012). Early complementopathy after multiple injuries in humans. Shock.

[19] Ward NS, Casserly B, Ayala A (2008). The Compensatory Anti-inflammatory Response Syndrome (CARS) in critically ill patients. Clin Chest Med.

[20] Appleton RT, Kinsella J, Quasim T (2015). The incidence of intensive care unit-acquired weakness syndromes: a systematic review. J Intensive Care Soc.

[21] Mira JC, Brakenridge SC, Moldawer LL, Moore FA (2017). Persistent inflammation, immunosuppression and catabolism syndrome. Crit Care Clin.

[22] Hodgson CL, Tipping CJ (2017). Physiotherapy management of intensive care unit-acquired weakness. Aust J Phys.

[23] Puthucheary ZA, Rawal J, McPhail M, Connolly B, Ratnayake G, Chan P (2013). Acute skeletal muscle wasting in critical illness. JAMA.

[24] Wafaisade A, Lefering R, Bouillon B, Sakka SG, Thamm OC, Paffrath T (2011). Epidemiology and risk factors of sepsis after multiple trauma: an analysis of 29,829 patients from the trauma registry of the German Society for Trauma Surgery. Crit Care Med.

[25] Liu T, Xie J, Yang F, Chen J-J, Li Z-F, Yi C-L (2015). The influence of sex on outcomes in trauma patients: a meta-analysis. Am J Surg.

[26] Verma P, Bhoi S, Baitha U, Sinha TP, Mishra PR (2017). Gender-based assessment of survival in trauma-hemorrhagic shock:a retrospective analysis of Indian population. Indian J Crit Care Med.

[27] Hicks CW, Hashmi ZG, Velopulos C, Efron DT, Schneider EB, Haut ER (2014). Association between race and age in survival after trauma. JAMA Surg.

[28] Rainey WE, Carr BR, Sasano H, Suzuki T, Mason JI (2002). Dissecting human adrenal androgen production. Trends Endocrinol Metab.

[29] Orentreich N, Brind JL, Vogelman JH, Andres R, Baldwin H (1992). Long-term longitudinal measurements of plasma dehydroepiandrosterone sulfate in normal men. J Clin Endocrinol Metab.

[30] Saltzman E, Guay A (2006). Dehydroepiandrosterone therapy as female androgen replacement. Semin Reprod Med.

[31] Rege J, Rainey WE (2012). The steroid metabolome of adrenarche. J Endocrinol.

[32] Labrie F, Diamond P, Cusan L, Gomez JL, Bélanger A, Candas B (1997). Effect of 12-month dehydroepiandrosterone replacement therapy on bone, vagina, and endometrium in postmenopausal women. J Clin Endocrinol Metab.

[33] Kiechl S, Willeit J, Bonora E, Schwarz S, Xu Q (2000). No association between dehydroepiandrosterone sulfate and development of atherosclerosis in a prospective population study (Bruneck Study). Arterioscler Thromb Vasc Biol.

[34] Jarrar D, Kuebler JF, Wang P, Bland KI, Chaudry IH (2001). DHEA: a novel adjunct for the treatment of male trauma patients. Trends Mol Med.

[35] Rainey WE, Nakamura Y (2008). Regulation of the adrenal androgen biosynthesis. J Steroid Biochem Mol Biol.

[36] Krug A, Ziegler C, Bornstein S, Ritsner MS, Weizman A (2008). DHEA and DHEA-S, and their functions in the brain and adrenal medulla. Neuroactive steroids in brain function, behavior and neuropsychiatric disorders.

[37] Ghosh D (2007). Human sulfatases: a structural perspective to catalysis. Cell Mol Life Sci.

[38] Mueller JW, Gilligan LC, Idkowiak J, Arlt W, Foster PA (2015). The regulation of steroid action by sulfation and desulfation. Endocr Rev.

[39] Prough RA, Clark BJ, Klinge CM (2016). Novel mechanisms for DHEA action. J Mol Endocrinol.

[40] Svec F, Porter JR (1998). The actions of exogenous dehydroepiandrosterone in experimental animals and humans. Proc Soc Exp Biol Med.

[41] Hazeldine J, Arlt W, Lord JM (2010). Dehydroepiandrosterone as a regulator of immune cell function. J Steroid Biochem Mol Biol.

[42] Radford DJ, Wang K, McNelis JC, Taylor AE, Hechenberger G, Hofmann J (2010). Dehydroepiandrosterone sulfate directly activates protein kinase C-beta to increase human neutrophil superoxide generation. Mol Endocrinol.

[43] Bancos I, Hazeldine J, Chortis V, Hampson P, Taylor AE, Lord JM (2017). Primary adrenal insufficiency is associated with impaired natural killer cell function: a potential link to increased mortality. Eur J Endocrinol.

[44] Smans LCCJ, Souverein PC, Leufkens HGM, Hoepelman AIM, Zelissen PMJ (2013). Increased use of antimicrobial agents and hospital admission for infections in patients with primary adrenal insufficiency: a cohort study. Eur J Endocrinol.

[45] Edvardsen Kine, Bjånesøy Trine, Hellesen Alexander, Breivik Lars, Bakke Marit, Husebye Eystein S., Bratland Eirik (2015). Peripheral Blood Cells from Patients with Autoimmune Addison's Disease Poorly Respond to Interferons In Vitro, Despite Elevated Serum Levels of Interferon-Inducible Chemokines. Journal of Interferon & Cytokine Research.

[46] Parker LN, Levin ER, Lifrak ET (1985). Evidence for adrenocortical adaptation to severe illness. J Clin Endocrinol Metab.

[47] Phillips AC, Carroll D, Gale CR, Lord JM, Arlt W, Batty GD (2010). Cortisol, DHEA sulphate, their ratio, and all-cause and cause-specific mortality in the Vietnam Experience Study. Eur J Endocrinol.

[48] Carroll D, Phillips AC, Lord JM, Arlt W, Batty GD (2011). Cortisol, dehydroepiandrosterone sulphate, their ratio and hypertension: evidence of associations in male veterans from the Vietnam Experience Study. J Hum Hypertens.

[49] Phillips AC, Upton J, Duggal NA, Carroll D, Lord JM (2013). Depression following hip fracture is associated with increased physical frailty in older adults: the role of the cortisol: dehydroepiandrosterone sulphate ratio. BMC Geriatr BioMed Central.

[50] Duggal NA, Upton J, Phillips AC, Hampson P, Lord JM (2013). Depressive symptoms are associated with reduced neutrophil function in hip fracture patients. Brain Behav Immun.

[51] Duggal NA, Beswetherick A, Upton J, Hampson P, Phillips AC, Lord JM (2014). Depressive symptoms in hip fracture patients are associated with reduced monocyte superoxide production. Exp Gerontol.

[52] Hampson P, Foster M, Taylor A, Bentley C, Fallowfield J, Midwinter M, et al. The immune-endocrine mechanisms of trauma-induced sarcopenia. Endocr Abstr. 2014. 10.1530/endoabs.34.S5.1.

[53] Foster MA, Taylor AE, Hill NE, Bentley C, Bishop J, Bion JF, et al. The endocrine and metabolic response in male survivors of major trauma. bioRxiv. 2019. 10.1101/577502.

[54] Maninger N, Wolkowitz OM, Reus VI, Epel ES, Mellon SH (2009). Neurobiological and neuropsychiatric effects of dehydroepiandrosterone (DHEA) and DHEA sulfate (DHEAS). Front Neuroendocrinol.

[55] Mocking RJT, Pellikaan CM, Lok A, Assies J, Ruhé HG, Koeter MW (2015). DHEAS and cortisol/DHEAS-ratio in recurrent depression: state, or trait predicting 10-year recurrence?. Psychoneuroendocrinology.

[56] Kolditz M, Halank M, Schulte-Hubbert B, Höffken G (2010). Adrenal function is related to prognosis in moderate community-acquired pneumonia. Eur Respir J.

[57] Sharshar T, Bastuji-Garin S, De Jonghe B, Stevens RD, Polito A, Maxime V (2010). Hormonal status and ICU-acquired paresis in critically ill patients. Intensive Care Med.

[58] Sharshar T, Bastuji-Garin S, Polito A, De Jonghe B, Stevens RD, Maxime V (2011). Hormonal status in protracted critical illness and in-hospital mortality. Crit Care BioMed Central.

[59] Klouche K, Da Mota EF, Durant R, Amigues L, Corne P, Jonquet O (2007). Hypothalamic-pituitary-adrenal axis reactivity and dehydroepiandrosterone sulfate plasma concentrations in the critically ill elderly. Age Ageing.

[60] Chinga-Alayo E, Villena J, Evans AT, Zimic M (2005). Thyroid hormone levels improve the prediction of mortality among patients admitted to the intensive care unit. Intensive Care Med.

[61] Dimopoulou I, Stamoulis K, Ilias I, Tzanela M, Lyberopoulos P, Orfanos S (2007). A prospective study on adrenal cortex responses and outcome prediction in acute critical illness: results from a large cohort of 203 mixed ICU patients. Intensive Care Med.

[62] Beishuizen A, Thijs LG, Vermes I (2002). Decreased levels of dehydroepiandrosterone sulphate in severe critical illness: a sign of exhausted adrenal reserve?. Crit Care.

[63] Van den Berghe G, Baxter RC, Weekers F, Wouters P, Bowers CY, Iranmanesh A (2002). The combined administration of GH-releasing peptide-2 (GHRP-2), TRH and GnRH to men with prolonged critical illness evokes superior endocrine and metabolic effects compared to treatment with GHRP-2 alone. Clin Endocrinol.

[64] Spratt DI, Longcope C, Cox PM, Bigos ST, Wilbur-Welling C (1993). Differential changes in serum concentrations of androgens and estrogens (in relation with cortisol) in postmenopausal women with acute illness. J Clin Endocrinol Metab.

[65] Gottschlich MM, Khoury J, Warden GD, Kagan RJ (2009). An evaluation of the neuroendocrine response to sleep in pediatric burn patients. JPEN-Parenter Enter.

[66] Van Den Berghe G, Dezegher F, Wouters P, Schetz M, Verwaest C, Ferdinande P (1995). Dehydroepiandrosterone-sulfate in critical illness - effect of dopamine. Clin Endocrinol.

[67] Osorio A, Vara-Thorbeck R, Rosell J, Osorio C, Ortega E, Ruiz-Requena E (2002). Dehydroepiandrosterone sulfate and growth axis hormones in patients after surgery. World J Surg.

[68] Almoosa KF, Gupta A, Pedroza C, Watts NB (2014). Low testosterone levels are frequent in patients with acute respiratory failure and are associated with poor outcomes. Endocr Pract.

[69] Dolecek R, Tymonová J, Adámková M, Kadlcík M, Pohlídal A, Závodná R (2003). Endocrine changes after burns: the bone involvement. Acta Chir Plast.

[70] Dossett LA, Swenson BR, Heffernan D, Bonatti H, Metzger R, Sawyer RG (2008). High levels of endogenous estrogens are associated with death in the critically injured adult. J Trauma.

[71] Folan MM, Stone RA, Pittenger AL, Stoffel JA, Hess MM, Kroboth PD (2001). Dehydroepiandrosterone, dehydroepiandrosterone-sulfate, and cortisol concentrations in intensive care unit patients. Crit Care Med.

[72] Brorsson C, Dahlqvist P, Nilsson L, Thunberg J, Sylvan A, Naredi S (2014). Adrenal response after trauma is affected by time after trauma and sedative/analgesic drugs. Injury.

[73] Mueller Cornelia, Blum Claudine A., Trummler Michael, Stolz Daiana, Bingisser Roland, Mueller Christian, Tamm Michael, Mueller Beat, Schuetz Philipp, Christ-Crain Mirjam (2014). Association of Adrenal Function and Disease Severity in Community-Acquired Pneumonia. PLoS ONE.

[74] Foster M, Taylor A, Hill N, Staruch R, O D, Bion J, et al. The endocrine response to severe trauma: the Steroids and Immunity from injury to Rehabilitation (SIR) study. Endocr Abstr. 2014. 10.1530/endoabs.34.P367.

[75] Taylor AE, Keevil B, Huhtaniemi IT (2015). Mass spectrometry and immunoassay: how to measure steroid hormones today and tomorrow. Eur J Endocrinol.

[76] Butcher SK, Killampalli V, Lascelles D, Wang K, Alpar EK, Lord JM (2005). Raised cortisol: DHEAS ratios in the elderly after injury: potential impact upon neutrophil function and immunity. Aging Cell.

[77] Sato K, Iemitsu M, Aizawa K, Mesaki N, Fujita S (2011). Increased muscular dehydroepiandrosterone levels are associated with improved hyperglycemia in obese rats. Am J Physiol Endocrinol Metab.

[78] Angele MK, Catania RA, Ayala A, Cioffi WG, Bland KI, Chaudry IH (1998). Dehydroepiandrosterone: an inexpensive steroid hormone that decreases the mortality due to sepsis following trauma-induced hemorrhage. Arch Surg.

[79] Karishma KK, Herbert J (2002). Dehydroepiandrosterone (DHEA) stimulates neurogenesis in the hippocampus of the rat, promotes survival of newly formed neurons and prevents corticosterone-induced suppression. Eur J Neurosci.

[80] Lohman R, Yowell R, Barton S, Araneo B, Siemionow M (1997). Dehydroepiandrosterone protects muscle flap microcirculatory hemodynamics from ischemia/reperfusion injury: an experimental in vivo study. J Trauma.

[81] Punjabi U, Deslypere JP, Verdonck L, Vermeulen A (1983). Androgen and precursor levels in serum and testes of adult rats under basal conditions and after hCG stimulation. J Steroid Biochem.

[82] Zhou Yingqiao, Kang Jian, Chen Di, Han Ningning, Ma Haitian (2015). Ample Evidence: Dehydroepiandrosterone (DHEA) Conversion into Activated Steroid Hormones Occurs in Adrenal and Ovary in Female Rat. PLOS ONE.

[83] Kobbe Philipp, Micansky Fabian, Lichte Philipp, Sellei Richard Martin, Pfeifer Roman, Dombroski Derek, Lefering Rolf, Pape Hans Christoph (2013). Increased morbidity and mortality after bilateral femoral shaft fractures: Myth or reality in the era of damage control?. Injury.

[84] Lichte P, Pfeifer R, Werner BE, Ewers P, Tohidnezhad M, Pufe T, et al. Dehydroepiandrosterone modulates the inflammatory response in a bilateral femoral shaft fracture model. Eur J Med Res. 2014;19(1):27. 10.1186/2047-783X-19-27 PMC4040478 24886543

[85] Knoferl MW, Angele MK, Catania RA, Diodato MD, Bland KI, Chaudry IH (2003). Immunomodulatory effects of dehydroepiandrosterone in proestrus female mice after trauma-hemorrhage. JAP.

[86] Catania RA, Angele MK, Ayala A, Cioffi WG, Bland KI, Chaudry IH (1999). Dehydroepiandrosterone restores immune function following trauma-haemorrhage by a direct effect on T lymphocytes. Cytokine.

[87] Oberbeck R, Deckert H, Bangen J, Kobbe P, Schmitz D (2007). Dehydroepiandrosterone: a modulator of cellular immunity and heat shock protein 70 production during polymicrobial sepsis. Intensive Care Med.

[88] Araneo BA, Shelby J, Li GZ, Ku W, Daynes RA (1993). Administration of dehydroepiandrosterone to burned mice preserves normal immunologic competence. Arch Surg..

[89] Araneo BA, Ryu SY, Barton S, Daynes RA (1995). Dehydroepiandrosterone reduces progressive dermal ischemia caused by thermal injury. J Surg Res.

[90] Hampson P, Dinsdale RJ, Wearn CM, Bamford AL, Bishop JRB, Hazeldine J (2017). Neutrophil dysfunction, immature granulocytes, and cell-free dna are early biomarkers of sepsis in burn-injured patients: a prospective observational cohort study. Ann Surg.

[91] Perner A, Nielsen SE, Rask-Madsen J (2003). High glucose impairs superoxide production from isolated blood neutrophils. Intensive Care Med.

[92] Corsini E, Galbiati V, Papale A, Kummer E, Pinto A, Serafini MM (2016). Role of androgens in dhea-induced rack1 expression and cytokine modulation in monocytes. Immun Ageing.

[93] Curatola Anna-Maria, Huang Kui, Naftolin Frederick (2012). Dehydroepiandrosterone (DHEA) Inhibition of Monocyte Binding by Vascular Endothelium Is Associated With Sialylation of Neural Cell Adhesion Molecule. Reproductive Sciences.

[94] Schmidt M, Kreutz M, Loffler G, Scholmerich J, Straub RH (2000). Conversion of dehydroepiandrosterone to downstream steroid hormones in macrophages. J Endocrinol.

[95] Li J, Chen J, Kirsner R (2007). Pathophysiology of acute wound healing. Clin Dermatol.

[96] Reinke J.M., Sorg H. (2012). Wound Repair and Regeneration. European Surgical Research.

[97] Ayhan S, Tugay C, Norton S, Araneo B, Siemionow M (2003). Dehydroepiandrosterone protects the microcirculation of muscle flaps from ischemia-reperfusion injury by reducing the expression of adhesion molecules. Plast Reconstr Surg.

[98] Shin MH, Rhie G-E, Park C-H, Kim KH, Cho KH, Eun HC (2005). Modulation of collagen metabolism by the topical application of dehydroepiandrosterone to human skin. J Invest Dermatol.

[99] Padgett David A, Loria Roger M (1998). Endocrine regulation of murine macrophage function: effects of dehydroepiandrosterone, androstenediol, and androstenetriol. Journal of Neuroimmunology.

[100] Cutolo M, Capellino S, Montagna P, Ghiorzo P, Sulli A, Villaggio B (2005). Sex hormone modulation of cell growth and apoptosis of the human monocytic/macrophage cell line. Arthritis Res Ther.

[101] Corcoran MP, Meydani M, Lichtenstein AH, Schaefer EJ, Dillard A, Lamon-Fava S (2010). Sex hormone modulation of proinflammatory cytokine and C-reactive protein expression in macrophages from older men and postmenopausal women. J Endocrinol.

[102] Mills SJ, Ashworth JJ, Gilliver SC, Hardman MJ, Ashcroft GS (2005). The sex steroid precursor DHEA accelerates cutaneous wound healing via the estrogen receptors. J Invest Dermatol.

[103] Head injury: triage, assessment, investigation and early management of head injury in children, young people and adults. National Institute for Health and Care Excellence (UK); 2014. https://www.nice.org.uk/guidance/cg176/chapter/Introduction. Accessed 21 July 2018. 25340248

[104] Stoffel-Wagner B (2001). Neurosteroid metabolism in the human brain. Eur J Endocrinol.

[105] Dong Y, Zheng P (2011). Dehydroepiandrosterone sulphate: action and mechanism in the brain. J Neuroendocrinol.

[106] Malik AS, Narayan RK, Wendling WW, Cole RW, Pashko LL, Schwartz AG (2003). A novel dehydroepiandrosterone analog improves functional recovery in a rat traumatic brain injury model. J Neurotrauma.

[107] Hoffman SW, Virmani S, Simkins RM, Stein DG (2003). The delayed administration of dehydroepiandrosterone sulfate improves recovery of function after traumatic brain injury in rats. J Neurotrauma Inc.

[108] Suzuki M, Wright LS, Marwah P, Lardy HA, Svendsen CN (2004). Mitotic and neurogenic effects of dehydroepiandrosterone (DHEA) on human neural stem cell cultures derived from the fetal cortex. Proc Natl Acad Sci.

[109] Rahmani Anahita, Shoae-Hassani Alireza, Keyhanvar Peyman, Kheradmand Danial, Darbandi-Azar Amir (2013). Dehydroepiandrosterone Stimulates Nerve Growth Factor and Brain Derived Neurotrophic Factor in Cortical Neurons. Advances in Pharmacological Sciences.

[110] Kibaly C, Meyer L, Patte-Mensah C, Mensah-Nyagan AG (2008). Biochemical and functional evidence for the control of pain mechanisms by dehydroepiandrosterone endogenously synthesized in the spinal cord. FASEB J.

[111] de Vries G-J, Olff M (2009). The lifetime prevalence of traumatic events and posttraumatic stress disorder in the Netherlands. J Trauma Stress.

[112] O'Donnell ML, Creamer M, Elliott P, Atkin C, Kossmann T (2005). Determinants of quality of life and role-related disability after injury: impact of acute psychological responses. J Trauma.

[113] Salyers MP, Evans LJ, Bond GR, Meyer PS (2004). Barriers to assessment and treatment of posttraumatic stress disorder and other trauma-related problems in people with severe mental illness: clinician perspectives. Community Ment Health J.

[114] Pitman RK, Rasmusson AM, Koenen KC, Shin LM, Orr SP, Gilbertson MW (2012). Biological studies of post-traumatic stress disorder. Nat Rev Neurosci.

[115] Michael A, Jenaway A, Paykel ES, Herbert J (2000). Altered salivary dehydroepiandrosterone levels in major depression in adults. Biol Psychiatry.

[116] Souza-Teodoro LH, de Oliveira C, Walters K, Carvalho LA (2016). Higher serum dehydroepiandrosterone sulfate protects against the onset of depression in the elderly: findings from the English Longitudinal Study of Aging (ELSA). Psychoneuroendocrinology.

[117] Grimley Evans J, Malouf R, Huppert F, van Niekerk JK. Dehydroepiandrosterone (DHEA) supplementation for cognitive function in healthy elderly people. Cochrane Database Syst Rev. 2006. 10.1002/14651858.CD006221. 10.1002/14651858.CD006221 PMC8988513 17054283

[118] Schmidt PJ, Daly RC, Bloch M, Smith MJ, Danaceau MA, St Clair LS (2005). Dehydroepiandrosterone monotherapy in midlife-onset major and minor depression. Arch Gen Psychiatry.

[119] Strous RD, Maayan R, Lapidus R, Stryjer R, Lustig M, Kotler M (2003). Dehydroepiandrosterone augmentation in the management of negative, depressive, and anxiety symptoms in schizophrenia. Arch Gen Psychiatry.

[120] Strous RD, Stryjer R, Maayan R, Gal G, Viglin D, Katz E (2007). Analysis of clinical symptomatology, extrapyramidal symptoms and neurocognitive dysfunction following dehydroepiandrosterone (DHEA) administration in olanzapine treated schizophrenia patients: a randomized, double-blind placebo controlled trial. Psychoneuroendocrinology.

[121] Peixoto C, Devicari Cheda JN, Nardi AE, Veras AB, Cardoso A (2014). The effects of dehydroepiandrosterone (DHEA) in the treatment of depression and depressive symptoms in other psychiatric and medical illnesses: a systematic review. Curr Drug Targets.

[122] Alkatib AA, Cosma M, Elamin MB, Erickson D, Swiglo BA, Erwin PJ (2009). A systematic review and meta-analysis of randomized placebo-controlled trials of DHEA treatment effects on quality of life in women with adrenal insufficiency. J Clin Endocrinol Metab.

[123] Genazzani AR, Inglese S, Lombardi I, Pieri M, Bernardi F, Genazzani AD (2004). Long-term low-dose dehydroepiandrosterone replacement therapy in aging males with partial androgen deficiency. Aging Male.

[124] Brooke AM, Kalingag LA, Miraki-Moud F, Camacho-Hübner C, Maher KT, Walker DM (2006). Dehydroepiandrosterone (DHEA) replacement reduces growth hormone (GH) dose requirement in female hypopituitary patients on GH replacement. Clin Endocrinol.

[125] Sripada RK, Marx CE, King AP, Rajaram N, Garfinkel SN, Abelson JL (2013). DHEA enhances emotion regulation neurocircuits and modulates memory for emotional stimuli. Neuropsychopharmacology.

[126] Yoon S-Y, Roh D-H, Seo H-S, Kang S-Y, Moon J-Y, Song S (2010). An increase in spinal dehydroepiandrosterone sulfate (DHEAS) enhances NMDA-induced pain via phosphorylation of the NR1 subunit in mice: involvement of the sigma-1 receptor. Neuropharmacology.

[127] Wang L, Wang Y-D, Wang W-J, Li D-J (2009). Differential regulation of dehydroepiandrosterone and estrogen on bone and uterus in ovariectomized mice. Osteoporos Int.

[128] Gentilini MV, Pesce Viglietti AI, Arriola Benitez PC, Iglesias Molli AE, Cerrone GE, Giambartolomei GH (2018). Inhibition of osteoblast function by Brucella abortusis reversed by dehydroepiandrosterone and involves ERK1/2 and estrogen receptor. Front Immunol Frontiers.

[129] Jankowski CM, Wolfe P, Schmiege SJ, Nair KS, Khosla S, Jensen M (2019). Sex-specific effects of dehydroepiandrosterone (DHEA) on bone mineral density and body composition: a pooled analysis of four clinical trials. Clin Endocrinol.

[130] Villareal DT (2002). Effects of dehydroepiandrosterone on bone mineral density: what implications for therapy?. Treat Endocrinol.

[131] Wang L, Wang Y-D, Wang W-J, Zhu Y, Li D-J (2007). Dehydroepiandrosterone improves murine osteoblast growth and bone tissue morphometry via mitogen-activated protein kinase signaling pathway independent of either androgen receptor or estrogen receptor. J Mol Endocrinol.

[132] Callies F, Fassnacht M, van Vlijmen JC, Koehler I, Huebler D, Seibel MJ (2001). Dehydroepiandrosterone replacement in women with adrenal insufficiency: effects on body composition, serum leptin, bone turnover, and exercise capacity. J Clin Endocrinol Metab.

[133] Dhatariya K, Bigelow ML, Nair KS (2005). Effect of dehydroepiandrosterone replacement on insulin sensitivity and lipids in hypoadrenal women. Diabetes.

[134] Gurnell EM, Hunt PJ, Curran SE, Conway CL, Pullenayegum EM, Huppert FA (2008). Long-term DHEA replacement in primary adrenal insufficiency: a randomized, controlled trial. J Clin Endocrinol Metab.

[135] Villareal DT, Holloszy JO, Kohrt WM (2000). Effects of DHEA replacement on bone mineral density and body composition in elderly women and men. Clin Endocrinol.

[136] Sun Y, Mao M, Sun L, Feng Y, Yang J, Shen P (2002). Treatment of osteoporosis in men using dehydroepiandrosterone sulfate. Chin Med J.

[137] Mühlen v D, Laughlin GA, Kritz-Silverstein D, Bergstrom J, Bettencourt R (2008). Effect of dehydroepiandrosterone supplementation on bone mineral density, bone markers, and body composition in older adults: the DAWN trial. Osteoporos Int.

[138] Jankowski CM, Gozansky WS, Schwartz RS, Dahl DJ, Kittelson JM, Scott SM (2006). Effects of dehydroepiandrosterone replacement therapy on bone mineral density in older adults: a randomized, controlled trial. J Clin Endocrinol Metab.

[139] Morales AJ, Haubrich RH, Hwang JY, Asakura H, Yen S (1998). The effect of six months treatment with a 100 mg daily dose of dehydroepiandrosterone (DHEA) on circulating sex steroids, body composition and muscle strength in age-advanced men and women. Clin Endocrinol.

[140] Yen SS, Morales AJ, Khorram O (1995). Replacement of DHEA in aging men and women. Potential remedial effects. Ann N Y Acad Sci.

[141] Villareal DT, Holloszy JO (2004). Effect of DHEA on abdominal fat and insulin action in elderly women and men: a randomized controlled trial. JAMA.

[142] van Thiel SW, Romijn JA, Pereira AM, Biermasz NR, Roelfsema F, van Hemert A (2005). Effects of dehydroepiandrostenedione, superimposed on growth hormone substitution, on quality of life and insulin-like growth factor I in patients with secondary adrenal insufficiency: a randomized, placebo-controlled, cross-over trial. J Clin Endocrinol Metab.

[143] Srinivasan M, Irving BA, Dhatariya K, Klaus KA, Hartman SJ, McConnell JP (2009). Effect of dehydroepiandrosterone replacement on lipoprotein profile in hypoadrenal women. J Clin Endocrinol Metab.

[144] Christiansen JJ, Gravholt CH, Fisker S, Møller N, Andersen M, Svenstrup B (2005). Very short term dehydroepiandrosterone treatment in female adrenal failure: impact on carbohydrate, lipid and protein metabolism. Eur J Endocrinol.

[145] Coutinho AE, Chapman KE (2011). The anti-inflammatory and immunosuppressive effects of glucocorticoids, recent developments and mechanistic insights. Mol Cell Endocrinol.

[146] Demling RH (1999). Comparison of the anabolic effects and complications of human growth hormone and the testosterone analog, oxandrolone, after severe burn injury. Burns.

[147] Reeves PT, Herndon DN, Tanksley JD, Jennings K, Klein GL, Micak RP (2016). Five-year outcomes after long-term oxandrolone administration in severely burned children: a randomized clinical trial. Shock.

[148] Gervasio JM, Dickerson RN, Swearingen J, Yates ME, Yuen C, Fabian TC (2000). Oxandrolone in trauma patients. Pharmacotherapy.

[149] Li H, Guo Y, Yang Z, Roy M, Guo Q (2016). The efficacy and safety of oxandrolone treatment for patients with severe burns: a systematic review and meta-analysis. Burns.

[150] Miller JT, Btaiche IF (2009). Oxandrolone treatment in adults with severe thermal injury. Pharmacotherapy.

[151] Johnson M, Ramey E, Ramwell PW (1975). Sex and age differences in human platelet aggregation. Nature.

[152] Drugs.com Database. Drugsite Trust, Auckland, NZ. https://www.drugs.com/mca/dhea. Accessed 14 February 2019.

[153] Muller M, van den Beld AW, Bots ML, Grobbee DE, Lamberts SWJ, van der Schouw YT (2004). Endogenous sex hormones and progression of carotid atherosclerosis in elderly men. Circulation.

[154] Merritt P, Stangl B, Hirshman E, Verbalis J (2012). Administration of dehydroepiandrosterone (DHEA) increases serum levels of androgens and estrogens but does not enhance short-term memory in post-menopausal women. Brain Res.

[155] Genazzani AD, Lanzoni C, Genazzani AR (2007). Might DHEA be considered a beneficial replacement therapy in the elderly?. Drugs Aging.

[156] Bowers LD (1999). Oral dehydroepiandrosterone supplementation can increase the testosterone/epitestosterone ratio. Clin Chem.

[157] Gingell JC, Knönagel H, Kurth KH, Tunn UW (1995). Placebo controlled double-blind study to test the efficacy of the aromatase inhibitor atamestane in patients with benign prostatic hyperplasia not requiring operation. The Schering 90.062 Study Group. J Urol.

[158] Forman EJ, Franasiak JM, Scott RT (2015). Elevated progesterone levels in women on DHEA supplementation likely represent assay interference. J Assist Reprod Genet.

[159] Elke G, Felbinger TW, Heyland DK (2015). Gastric residual volume in critically ill patients: a dead marker or still alive?. Nutr Clin Pract.

[160] Labrie F, Archer DF, Koltun W, Vachon A, Young D, Frenette L (2016). Efficacy of intravaginal dehydroepiandrosterone (DHEA) on moderate to severe dyspareunia and vaginal dryness, symptoms of vulvovaginal atrophy, and of the genitourinary syndrome of menopause. Menopause.

[161] Labrie F, Cusan L, Gomez JL, Martel C, Bérubé R, Bélanger P (2008). Changes in serum DHEA and eleven of its metabolites during 12-month percutaneous administration of DHEA. J Steroid Biochem Mol Biol.

[162] Li H, Klein G, Sun P, Buchan AM (2001). Dehydroepiandrosterone (DHEA) reduces neuronal injury in a rat model of global cerebral ischemia. Brain Res.

[163] Wren BG, Day RO, McLachlan AJ, Williams KM (2003). Pharmacokinetics of estradiol, progesterone, testosterone and dehydroepiandrosterone after transbuccal administration to postmenopausal women. Climacteric.

[164] Keane K, Hinchliffe P, Namdar N, Conceicao J, Newsholme P, Yovich J. Novel dehydroepiandrosterone troche supplementation improves the serum androgen profile of women undergoing in vitro fertilization. Drug Des Dev Ther. 2015;9:5569–78. 10.2147/DDDT.S92467 PMC4607057 26487801

[165] Scheffers CS, Armstrong S, Cantineau AE, Farquhar C, Jordan V. Dehydroepiandrosterone for women in the peri- or postmenopausal phase. Cochrane Db Syst Rev. 2015;1:CD011066. 10.1002/14651858.CD011066.pub2 PMC10662543 25879093

[166] Tummala S, Svec F (1999). Correlation between the administered dose of DHEA and serum levels of DHEA and DHEA-S in human volunteers: analysis of published data. Clin Biochem.

[167] Arlt W, Justl HG, Callies F, Reincke M, Hübler D, Oettel M (1998). Oral dehydroepiandrosterone for adrenal androgen replacement: pharmacokinetics and peripheral conversion to androgens and estrogens in young healthy females after dexamethasone suppression. J Clin Endocrinol Metab.

[168] Crosbie D, Black C, McIntyre L, Royle PL, Thomas S. Dehydroepiandrosterone for systemic lupus erythematosus. Cochrane Db Syst Rev. 2007;(4):CD005114. 10.1002/14651858.CD005114.pub2. 10.1002/14651858.CD005114.pub2 PMC8864970 17943841

[169] Nagels HE, Rishworth JR, Siristatidis CS, Kroon B. Androgens (dehydroepiandrosterone or testosterone) for women undergoing assisted reproduction. Cochrane Db Syst Rev. 2015. 10.1002/14651858.CD009749.pub2. 10.1002/14651858.CD009749.pub2 PMC10559340 26608695

[170] Gupta Bhawna, Mittal Preeti, Khuteta Rakesh, Bhargava Adarsh (2012). A Comparative Study of CEE, Tibolone, and DHEA as Hormone Replacement Therapy for Surgical Menopause. The Journal of Obstetrics and Gynecology of India.

[171] El-Alfy M, Deloche C, Azzi L, Bernard BA, Bernerd F, Coutet J (2010). Skin responses to topical dehydroepiandrosterone: implications in antiageing treatment?. Br J Dermatol..

[172] Forsblad-d'Elia H, Carlsten H, Labrie F, Konttinen YT, Ohlsson C (2009). Low serum levels of sex steroids are associated with disease characteristics in primary Sjogren's syndrome; supplementation with dehydroepiandrosterone restores the concentrations. J Clin Endocrinol Metab.

[173] Nestler JE, Barlascini CO, Clore JN, Blackard WG (1988). Dehydroepiandrosterone reduces serum low density lipoprotein levels and body fat but does not alter insulin sensitivity in normal men. J Clin Endocrinol Metab.

[174] Nair KS, Rizza RA, O'Brien P, Dhatariya K, Short KR, Nehra A (2006). DHEA in elderly women and DHEA or testosterone in elderly men. N Engl J Med.

[175] Vogiatzi MG, Boeck MA, Vlachopapadopoulou E, El-Rashid R, New MI (1996). Dehydroepiandrosterone in morbidly obese adolescents: effects on weight, body composition, lipids, and insulin resistance. Metabolism.

[176] Piketty C, Jayle D, Leplege A, Castiel P, Ecosse E, Gonzalez-Canali G (2001). Double-blind placebo-controlled trial of oral dehydroepiandrosterone in patients with advanced HIV disease. Clin Endocrinol.

